# Increased carotid artery intima-media thickness and myeloperoxidase level in children with newly diagnosed juvenile idiopathic arthritis

**DOI:** 10.1186/s13075-015-0699-x

**Published:** 2015-07-16

**Authors:** Jaanika Ilisson, Maksim Zagura, Kersti Zilmer, Erik Salum, Kaire Heilman, Anneli Piir, Vallo Tillmann, Jaak Kals, Mihkel Zilmer, Chris Pruunsild

**Affiliations:** Department of Paediatrics, Faculty of Medicine, University of Tartu, Lunini 6, Tartu, 51014 Estonia; Children’s Clinic, Tartu University Hospital, Tartu, Estonia; Department of Biochemistry, Institute of Biomedicine and Translational Medicine, The Centre of Excellence for Translational Medicine, Faculty of Medicine, University of Tartu, Tartu, Estonia; Department of Cardiology, Faculty of Medicine, University of Tartu, Tartu, Estonia; Tallinn Children’s Hospital, Tallinn, Estonia; Department of Vascular Surgery, Tartu University Hospital, Tartu, Estonia

## Abstract

**Introduction:**

Juvenile idiopathic arthritis (JIA) is a frequent childhood rheumatic disease characterized by chronic inflammation. The latter has been related to impairment of arterial functional-structural properties, atherogenesis and later cardiovascular events. The objective of this study was to examine intima-media thickness (IMT) and the parameters of arterial stiffness in children with JIA at diagnosis and their correlation with JIA subtype and markers of inflammation and atherosclerosis.

**Methods:**

Thirty-nine newly diagnosed patients with JIA (26 girls; mean age, 13.2 ± 2.6 years) and 27 healthy controls (9 girls; mean age, 13.6 ± 3.4 years) were included in the study. Twelve patients had oligoarthritis, fifteen had extended oligoarthritis and twelve had rheumatoid factor–negative polyarthritis. IMT of the common carotid artery was determined by ultrasonography, carotid-femoral pulse wave velocity (cfPWV) and augmentation index adjusted to a heart rate of 75 beats/min (AIx@75) were determined by applanation tonometry. The serum levels of atherosclerosis-related biomarkers, such as asymmetric dimethylarginine (ADMA), myeloperoxidase (MPO) and adiponectin, were measured by enzyme-linked immunosorbent assay.

**Results:**

Mean IMT (0.46 ± 0.04 vs. 0.42 ± 0.04 mm; *p* = 0.0003) and MPO concentration (115.2 [95 % confidence interval {95 % CI}, 97.4–136.3] vs. 57.6 [95 % CI, 47.1–70.3] ng/ml; *p* < 0.0001) were higher in the patients with JIA than in the control subjects. The cfPWV, AIx@75 and serum ADMA and adiponectin levels did not significantly differ between the groups and JIA subtypes. Serum adiponectin level correlated negatively with AIx@75 in patients with JIA (*r* = −0.38; *p* < 0.05).

**Conclusions:**

Patients with JIA have increased mean IMT and elevated MPO levels at early stages of the disease. AIx@75 was inversely independently associated with adiponectin level in the patients, suggesting that lower adiponectin levels might influence arterial subclinical stiffening in patients with newly diagnosed JIA.

## Introduction

Juvenile idiopathic arthritis (JIA) is the most common chronic rheumatic disease in childhood, with an onset before the age of 16 years and a potential to persist into adulthood. JIA is a heterogeneous group of disorders with different disease progression and prognosis. Of the JIA subtypes, persistent oligoarthritis and monocyclic systemic arthritis have the best prognosis and polyarthritis the worst [1, 2]. Approximately 50 % of patients with JIA still have active disease and need for disease-modifying antirheumatic medications in adulthood [3–5].

Many patients with active JIA may not present with high levels of classical inflammatory markers such as C-reactive protein or erythrocyte sedimentation rate. Therefore, studies on the role of new markers (e.g., myeloperoxidase [MPO], asymmetric dimethylarginine [ADMA], adiponectin) may open some new aspects to describe inflammation in patients with JIA. MPO, ADMA and adiponectin have been linked to inflammation and/or oxidative stress [6–8]. Previous studies have shown that MPO levels are higher in patients with JIA compared with control groups [9]. ADMA and adiponectin have not been investigated in patients with JIA.

Atherosclerosis is considered an inflammatory disorder starting in childhood. Chronic inflammation has been suggested to play an important role in the developing of early atherosclerosis, which contributes to the development of cardiovascular disease (CVD) [10, 11].

CVD is more frequent in patients with rheumatoid arthritis (RA), and they are diagnosed at an earlier age than other patients with CVD. Mortality among RA patients is also higher than general population and is mostly due to cardiovascular diseases [12–14]. In addition to traditional risk factors for CVD, patients with a chronic inflammatory disease also have many disease-specific and drug-related risk factors [13]. Recent studies have shown that patients with RA have increased aortic stiffness, and this is correlated with markers of inflammation and aortic inflammation without any clinical signs of CVD [15, 16].

As JIA is a chronic inflammatory disease, it has long-term effects on many organ systems, including the cardiovascular system. Accordingly, it has been shown that patients with JIA have a moderate risk for premature cardiovascular events [17].

To date, only a few studies of vascular function, structural and atherosclerosis-related biomarkers in patients with JIA have been done. They have shown impaired endothelial dysfunction and alterations in vascular function compared with controls during the course of the disease [18, 19]. Satija et al. reported altered arterial wall indices in newly diagnosed patients with JIA, indicating increased arterial stiffness [20].

The aim of the present study was to evaluate intima-media thickness (IMT) and the parameters of arterial stiffness in patients with JIA at the time of diagnosis and to assess their correlation with JIA subtype and serum markers of inflammation and atherosclerosis.

## Methods

### Patients and controls

This cross-sectional study included 39 children with JIA (26 girls; mean age, 13.2 ± 2.6 years) who were patients of the paediatric rheumatologist at the outpatient children’s clinic of Tartu University Hospital, Estonia, and 27 healthy control subjects (9 girls; mean age, 13.6 ± 3.4 years) recruited from a previous diabetes study [21]. The study group included children aged 7–18 years with newly diagnosed JIA (onset of symptoms before the 16th birthday). JIA was diagnosed according to the International League of Associations for Rheumatology criteria [22]. Twenty-seven patients had oligoarthritis (fifteen of them had extended oligoarthritis), and twelve had rheumatoid factor (RF)-negative polyarthritis. All patients were RF-negative, three were antinuclear antibody–positive and five were human leukocyte antigen B27–positive.

Healthy control subjects were defined as without known chronical illness at the time of enrolment, without acute infection and no history of using antihypertensive or lipid-lowering medications. They were matched with the patients by age (±2 years). Three of the subjects from the original control group were excluded because of greater age difference. Written informed consent was obtained from each participant and the parent or legal representative. The study was approved by the ethics committee of Tartu University.

### Clinical and laboratory investigations

A full joint examination was performed by the same paediatric rheumatologist during the outpatient visit. Blood samples were obtained for nine different biochemical markers: glucose, creatinine, total cholesterol, high-density lipoprotein (HDL) cholesterol, low-density lipoprotein (LDL) cholesterol, triglycerides, ADMA, adiponectin and MPO. The first six markers are regularly used in clinical practice and were analysed in the local clinical laboratory using certified standard assays. For the last three markers, serum and plasma were stored at −80 °C until analysis. Serum ADMA concentration was determined by using a validated enzyme-linked immunosorbent assay (ELISA) kit (DLD Gesellschaft für Diagnostika und medizinische Geräte mbH, Hamburg, Germany). Serum adiponectin concentration was determined using the Human Adiponectin/Acrp30 Quantikine ELISA kit (R&D Systems Europe, Abingdon, UK). The adiponectin intraassay coefficient of variation (CV) is 4.6 %, and the interassay CV is 7.9 %. MPO was determined in serum of patients with JIA using an enzyme immunoassay test kit (catalogue number BC-1129; BioCheck, Foster City, CA, USA) and in plasma of controls using an ELISA kit (BIOXYTECH enzyme immunoassay for MPO, catalogue number 21013; OXIS Health Products, Portland, OR, USA), according to the manufacturer’s instructions. MPO intraassay CV is 5.7 % and inter-assay CV 8.5 %.

### Haemodynamic measurements

Brachial blood pressure (BP) and heart rate were recorded from the left arm with the subject in the supine position. BP was measured with an automated digital oscillometric BP monitor (OMRON M4-I; Omron Healthcare Europe, Hoofddorp, the Netherlands). Radial arterial pressure waveforms were obtained with a high-fidelity applanation tonometer (SPT-301B; Millar, Houston, TX, USA) applied to the wrist of the left hand. Pulse wave analysis (SCOR-Px 7.0; AtCor Medical, West Ryde, Australia) was then performed to generate a corresponding central (ascending aortic) waveform using a generalized transfer function, which has been prospectively validated for assessment of ascending aortic BP [23]. Augmentation index (AIx), mean arterial pressure (MAP), central systolic blood pressure, central diastolic blood pressure and central pulse pressure were determined by pulse wave analysis. The AIx was calculated as the difference between the second and the first systolic peaks, divided by pulse pressure and expressed in percentages [24]. The AIx values were adjusted to a heart rate of 75 beats/min (AIx@75) using a built-in algorithm in the SphygmoCor Px system (AtCor Medical). The within- and between-observer CVs for AIx@75 were 3.4 % and 7.1 %, respectively. Pulse wave velocity was measured by the foot-to-foot method using the same device. The aortic pulse wave velocity (aPWV) was determined by sequentially recording electrocardiogram-gated carotid and femoral artery waveforms, as described in detail previously [24]. The within- and between-observer CVs for aPWV were 2.3 % and 6.2 %, respectively. All measurements were made in duplicate by two trained investigators, and the mean values were used in subsequent analysis.

### Assessment of carotid intima-media thickness

Carotid ultrasound for evaluation of IMT was performed using the SonoSite M-Turbo portable ultrasound device (SonoSite, Bothell, WA, USA) coupled to a 5- to 10-MHz multifrequency high-resolution linear transducer. SonoCalc software (SonoSite) was used for calculation of mean IMT values. The measurements were obtained with the subject lying down, with the head extended and slightly turned opposite to the examined carotid. Measurements were made of the common carotid artery after the examination of a longitudinal section of 10 mm at a distance of 1 cm from the bifurcation. The measurements were performed in the far wall in the lateral, anterior and posterior projections. The within- and between-observer CVs for mean IMT were 2.2 % and 5.9 %, respectively.

A standard deviation score (SDS) of IMT for each subject was calculated using height-specific IMT normative values published by Jourdan et al. [25].

### Statistical analysis

Differences between the JIA and control groups were studied with analysis of variance, adjusted by sex. Because the distributions of some variables were not normally distributed, they were log-transformed for analysis as required to improve normality. The normally distributed data are presented as mean ± standard deviation; the non-normally distributed data are presented as the geometric mean with the 95 % confidence interval (CI). Multiple regression analysis was performed to investigate the independent determinants of AIx@75. To examine the associations between the variables, Pearson’s correlation test was used.

We used AIx@75 as a base for power calculations for *t* tests. A total of 66 persons were targeted to enter this two-group study. The probability is 80 % that the study will detect an effect size (the smallest difference between the means) at a two-sided 0.05 significance level if the true difference between groups is 4.953 U. This is based on the assumption that the standard deviation of the response variable is 10 [26]. Differences were considered statistically different if the *p* value was less than 0.05. Statistical analysis was performed using the SAS version 9.2 statistical software package (SAS Institute, Cary, NC, USA).

## Results

The subjects’ clinical and laboratory characteristics, as well as the arterial functional-structural properties of the study groups, are presented in Table 1.

**Table 1:** Clinical and laboratory data and arterial functional-structural properties in children with juvenile idiopathic arthritis and controls, adjusted by sex

Parameters	JIA group (*n* = 39)	Control group (*n* = 27)	*p* value
Age (yr)	13.0 ± 3.11	13.5 ± 3.08	0.5687
Body mass index (kg/m^2^)	21.2 ± 4.09	20.2 ± 4.04	0.3364
Peripheral systolic blood pressure (mmHg)	112.2 ± 8.7	108.9 ± 8.6	0.1375
Peripheral diastolic blood pressure (mmHg)	57.6 ± 5.01	57.1 ± 4.96	0.7360
Mean arterial blood pressure (mmHg)	74.0 ± 5.82	73.3 ± 5.77	0.6549
Central systolic blood pressure (mmHg)	92.0 ± 7.5	90.17 ± 7.4	0.3748
Central diastolic blood pressure (mmHg)	58.5 ± 5.07	58.4 ± 5.04	0.9249
Cholesterol (mmol/L)^a^	4.1 (3.8–4.3)	4.1 (3.8–4.4)	0.8150
LDL cholesterol (mmol/L)^a^	2.24 (2.06–2.43)	2.36 (2.13–2.60)	0.4324
HDL cholesterol (mmol/L)	1.50 ± 0.40	1.71 ± 0.40	0.0415
Triglycerides (mmol/L)^a^	0.87 (0.75–1.02)	0.70 (0.59–0.84)	0.0688
ADMA (μmol/L)	0.622 ± 0.175	0.679 ± 0.173	0.2040
MPO (ng/ml)^a^	115.2 (97.4–136.3)	57.6 (47.1–70.3)	<0.0001
Adiponectin (ng/ml)^a^	8596.7 (7296.3–10,128.9)	9123.1 (7506.6–11,087.7)	0.6519
Mean IMT (mm)	0.46 ± 0.04	0.42 ± 0.04	0.0003
IMT SDS, height-specific^b^	1.694 ± 0.858	0.744 ± 0.852	0.0002
cfPWV (m/s)^a^	4.92 (4.71–5.14)	5.18 (4.90–5.47)	0.1579
AIx@75 (%)	0.642 ± 11.258	−2.042 ± 11.179	0.3811

*ADMA* Asymmetric dimethylarginine, *AIx@75* augmentation index values adjusted to a heart rate of 75 beats/min, *cfPWV* carotid-femoral pulse wave velocity, *HDL* high-density lipoprotein cholesterol, *JIA* juvenile idiopathic arthritis, *LDL* low-density lipoprotein cholesterol, *MPO* myeloperoxidase, *SDS* standard deviation score

Values are mean ± standard deviation unless otherwise indicated

^a^Data were log-transformed before statistical analysis and are presented as geometric mean (95 % confidence interval)

^b^Data for34 patents with JIA and 23 control were analysed as reference values and are available for the height of 140 cm and taller [25]

The total cholesterol, LDL cholesterol, HDL cholesterol and triglyceride levels did not differ between groups. The same was found for serum ADMA and adiponectin levels (Table 1).

The serum level of MPO was significantly higher in patients with JIA compared with the plasma MPO concentrations in control subjects (115.2 [95 % CI, 97.4–136.3] vs. 57.6 [95 % CI, 47.1–70.3] ng/ml; *p* < 0.0001).

Mean IMT values and IMT height specific SDS were higher in the patients with JIA than in the control subjects (0.46 ± 0.04 vs. 0.42 ± 0.04 mm, *p* < 0.0003; 1.694 ± 0.858 vs. 0.744 ± 0.852, *p* = 0.0002). We did not find statistically significant differences between the groups with regard to carotid-femoral pulse wave velocity (cfPWV) or AIx@75 (Table 1).

Serum ADMA and adiponectin levels, as well as the mean IMT, cfPWV and AIx@75, did not differ significantly between the JIA subtypes (data not shown).

**Fig. 1:**
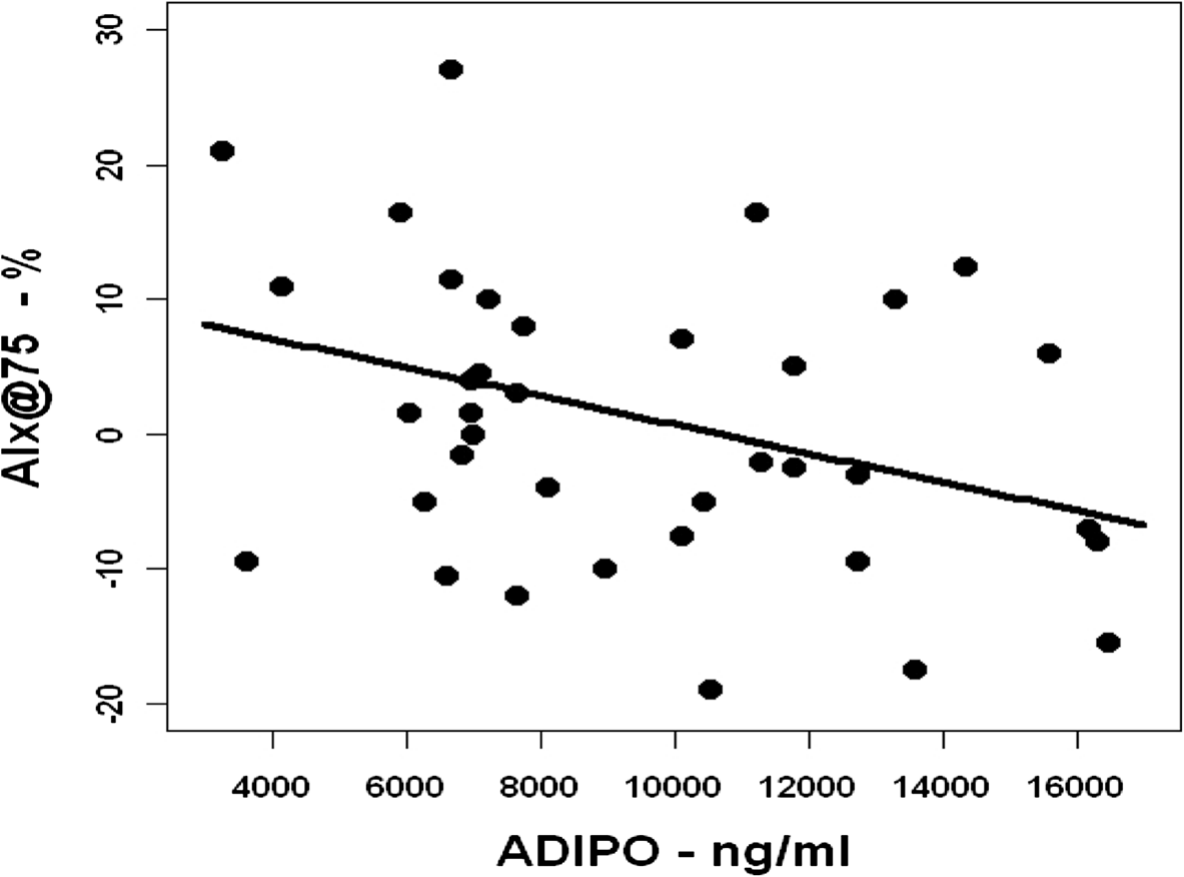
Correlation between serum adiponectin (ADIPO) concentration and augmentation index values adjusted to a heart rate of 75 beats/min (AIx@75) in patients with juvenile idiopathic arthritis (*r* = −0.38; *p* < 0.05)

There was a significant negative correlation between serum adiponectin level and AIx@75 in patients with JIA (*r* = −0.38; *p* < 0.05) (Fig. 1). No other statistically significant correlations were found.

In multiple regression analysis, AIx@75 was independently determined by age, MAP and adiponectin levels in patients with JIA, accounting for 29 % of its variability (*R*^2^(adj) = 0.29; *p* < 0.01) (Table 2).

**Table 2:** Multiple regression model for patients with juvenile idiopathic arthritis with augmentation index values adjusted to a heart rate of 75 beats/min as the dependent variable

Parameter	Regression coefficient	Standard deviation	*p* value
Age	−1.71	0.62	0.009
MAP	0.73	0.27	0.01
Adiponectin	−0.001	0.0004	0.03

*MAP* mean arterial pressure

## Discussion

We have demonstrated that, already at the time of JIA diagnosis, there are atherosclerosis-related changes in the arterial wall, supporting the detrimental effect of systemic inflammation on the cardiovascular system. The present study is the first clinical analysis of the relationship between adiponectin and arterial stiffness in patients with JIA. We have shown that AIx@75 is inversely related to adiponectin levels in a cohort of patients with JIA. This finding suggests that adiponectin might influence arterial stiffening in the early stages of the disease. In addition, we demonstrated that serum MPO is significantly higher in patients with JIA. Thus, MPO may be a sensitive marker of systemic inflammation and oxidative stress in patients with newly diagnosed JIA.

To date, IMT in patients with JIA has been reported in a few studies, with controversial results. Breda et al. reported increased mean IMT in these patients [19], whereas Vlahos et al. did not find a statistically significant difference in IMT between their JIA and control groups [18]. Comparing mean IMT between JIA subtypes, Vlahos et al. found that a systemic arthritis (high inflammatory activity) group had a thicker intima-media layer compared with the control group and the oligoarticular group [18]. Both of these studies were done in patients with previously diagnosed JIA [18, 19]. By contrast, our study was conducted with patients at the very early stage of the disease, and we demonstrated that IMT is increased in patients with JIA already in the early phase. We did not find a significant difference in IMT thickness between the oligo- and polyarticular subtypes. So far, only Breda et al. have investigated the changes in IMT longitudinally in patients with JIA. They found that after a 12-month treatment period, the mean IMT improved; that is, was less than at the start of the study [19]. This points out the importance of early, adequate, aggressive treatment of JIA to controlling the possibility of future cardiovascular events. Future investigations are needed to verify this point.

Several studies have shown that increased MPO concentration is associated with an increased risk of CVD [27, 28]. Also, studies have shown higher serum and plasma concentrations in patients with rheumatologic diseases [9, 29]. In our study, we found higher serum levels of MPO in patients with JIA already at an early stage of the disease. Thus, MPO may be a good marker of the level of inflammation, even when the classical inflammation markers are at normal levels.

ADMA acts as a natural inhibitor of nitric oxide synthase by reducing nitric oxide production. Higher levels of ADMA give rise to endothelial dysfunction and arterial stiffening [30]. The key enzyme in degradation of ADMA is dimethylarginine dimethylaminohydrolase, and its activity is downregulated by tumour necrosis factor α and has a known role in the pathogenesis of RA and JIA [31]. Higher levels of ADMA and its correlation to oxidative stress markers have been shown in patients with RA [7]. In children with other chronic diseases, such as chronic kidney disease or type 1 diabetes mellitus, elevated serum ADMA concentrations have been found [21, 32]. However, all these children already had their disease for many years. To our knowledge, the level of ADMA has not been investigated to date in patients with JIA. In our study, we did not find a significant difference in serum ADMA levels at the disease start between our patient and control groups.

Adiponectin has well-known anti-inflammatory properties. To the contrary, there are studies suggesting that hypoadiponectemia might play an important role in CVDs (e.g., hypertension) [33]. In patients with RA, adiponectin levels are higher than in patients with non-inflammatory conditions (e.g., osteoarthrosis), which is suggestive of a proinflammatory effect [8]. Alternatively, increased adiponectin might represent a compensatory response limiting further vascular injury [34]. To our knowledge, adiponectin levels have not been investigated in patients with JIA. In our study, we did not find statistically significant differences in levels of adiponectin between patients and controls, but adiponectin was inversely independently correlated with AIx@75 in patients with JIA, indicating that lower levels of adiponectin might be related to arterial stiffening already at an early stage of JIA.

In our study, there was no significant difference in cfPWV between the patients and the control subjects. These findings could be due to the fact that we studied newly diagnosed patients with JIA, whereas aortic stiffening may occur at later stages of the disease. Consistent with this hypothesis, a recent study has shown that patients with JIA with long-term active disease have increased aortic stiffness compared with control subjects [35].

The limitations of our study are the use of a historical control group, the gender difference and the absence of patients from all the JIA subtypes (although the two subgroups presented are the most frequent ones in every study group of patients with JIA), which limits extrapolation of the risk of vascular dysfunction to all patients with JIA. At the same time, we present a detailed, complex overview of different markers of inflammation, oxidative stress and arterial structural-functional properties, which describes the stage of development of subclinical atherosclerosis in these patients. Another limitation of our study is the use of generalized transfer function, which has been validated only in adults.

## Conclusions

Our study shows that patients with JIA have signs of alterations in arterial structural properties already at the time of diagnosis of the disease. Independent inverse association between the atherosclerosis-related biomarker adiponectin and arterial stiffness suggests that hypoadiponectemia may influence vascular function in these patients. Further investigations are needed to clarify the risk of vascular dysfunction during the disease course and to identify children who are at increased risk of early development of atherosclerosis in adulthood.

## Abbreviations

ADIPO: Adiponectin
ADMA: Asymmetric dimethylarginine
AIx: Augmentation index
AIx@75: Augmentation index values adjusted to a heart rate of 75 beats/min
aPWV: Aortic pulse wave velocity
BP: Blood pressure
cfPWV: Carotid-femoral pulse wave velocity
CI: Confidence interval
CV: Coefficient of variation
CVD: Cardiovascular disease
ELISA: Enzyme-linked immunosorbent assay
HDL: High-density lipoprotein
IMT: Intima-media thickness
JIA: Juvenile idiopathic arthritis
LDL: Low-density lipoprotein
MAP: Mean arterial pressure
MPO: Myeloperoxidase
RA: Rheumatoid arthritis
RF: Rheumatoid factor
SDS: Standard deviation score
